# The Microscopic Anatomy of the Teeth

**Published:** 1920-09

**Authors:** 


**The Microscopic Anatomy of the Teeth.** By J. HOWARD MUMMERY, D.Sc. Penn., M.R.C.S., L.D.S. Eng. Pp. viii., 382. London: Oxford Medical Publications. 1919. Price 25s. net.

Mr. Mummery's name has long been associated with research work on the structure and development of the dental tissues, and it is with very great pleasure that we welcome his latest publication. Nearly half the book is devoted to the enamel. The author is to be congratulated on being the first to describe the minute anatomy of this structure in the elephant. He confirms Smreker's views with regard to the enamel prisms, that they are not rounded or polygonal in transverse section, but have an arched form owing to the prisms being longitudinally grooved and fitting into one another. Perhaps Mr. Mummery is best known for his work on the innervation of the dentine, and it is here that his triumph is greatest. There seems little doubt, judging from his statements and his photographs, that the neurofibrils in the plexus beneath the odontoblasts do not pass direct to the dentine but enter small nerve-cells or corpuscles which lie in a row along the bases of the odontoblast cells. Each end cell contributes (1) an axon which passes up between the odontoblasts to enter the dentine with the dentinal fibril, (2) numerous dendrons which form a network around the odontoblasts. Another new feature of this book is the description of the sheath of Hertwig, which is shown to be not a continuation of the enamel organ, but a prolongation of the epithelial cells in the tooth follicle lying to its outer side. The illustrations, both plates and microphotographs, are exceedingly good, and the author expresses his own views clearly and convincingly, at the same time giving full credit to the findings of others.

